# Food Security of Adolescents in Selected Khat- and Coffee-Growing Areas in the Sidama Zone, Southern Ethiopia

**DOI:** 10.3390/nu10080980

**Published:** 2018-07-27

**Authors:** Denabo Billo Juju, Makiko Sekiyama, Osamu Saito

**Affiliations:** 1 United Nations University Institute for the Advanced Study of Sustainability, Tokyo 150-8925, Japan; juju@student.unu.edu; 2 National Institute for Environmental Studies, Tsukuba 305-0053, Japan; sekiyama.makiko@nies.go.jp

**Keywords:** adolescents, food security, stunting, thinness, khat, coffee, Ethiopia

## Abstract

Whilst pervasive food insecurity exists among adolescents in Ethiopia, the available information is scant and inconsistent. Therefore, the main objective of this cross-sectional study was to contribute to these gaps by assessing the food security of adolescents in the selected khat- and coffee-growing areas. We selected 234 (117 girls and 117 boys) adolescents aged 12–18 years via stratified random sampling. We measured the height and weight of the adolescents and asked about their food insecurity experiences. We assessed the prevalence of stunting and thinness using the WHO 2007 growth reference standards. Out of the total, 17 (7.3%) and 30 (12.8%) adolescents were stunted and thin, respectively. In addition, 89 (38.0%) adolescents reported food insecurity experiences and nine (3.8%) were overweight. A regression analyses showed that the stunting was associated with the age of the adolescents and maternal education. Thinness was associated with area, gender, and the number of meals. Food insecurity experiences were associated with health problems in the past 30 days. In general, adolescents from the khat-growing area have better food security than those from the coffee-growing area, and the same is true, gender-wise, for girls compared to boys. We recommend possible interventions primarily for adolescents in coffee-growing areas.

## 1. Introduction

Adolescents are defined as young people within the age range of 10–19 years and currently constitute the fastest growing segment of the population in most developing countries [1]. There were approximately 1.2 billion adolescents in the world in 2009, accounting for approximately 18% of the world population, and 88% of adolescents live in developing countries [2]. In 2016, nearly a quarter (24.8%) of Ethiopia’s population were adolescents aged 10–19 years [3]. A sizable proportion of adolescents from many developing countries, mostly in southeast Asia and Africa, suffer from undernutrition, which makes them highly vulnerable to potential diseases and premature death [4]. Their risk of vulnerability to undernutrition increases in situations where poverty, famine, and conflicts prevail [5]. Potential impacts of nutritional deficiencies such as stunting are difficult to deal with once adolescence is passed and will become permanent [6]. Catch up growths that occur during childhood and adolescence, usually after recovery from an illness or undernutrition, can restrain, or even correct, growth failures caused by acute malnutrition and chronic diseases [7]. Access to improved nutrition would help a stunted child or adolescent undergo a catch-up growth period and resume a normal growth trajectory before his/her height is permanently reduced [8].

Adolescence is a stage of growth marked by rapid physical growth (e.g., height), often known as an adolescent growth spurt, and behavioral changes [9]. It is at this stage that humans attain more than 20% of their adult height and gain up to 50% of their adult weight and skeletal mass [10]. The requirements for both energy and protein considerably increase during adolescence [4]. However, adolescents are often considered to be fit and less vulnerable to diseases compared to other segments of the society, such as children and the elderly and, therefore, the condition of their health has received little attention until recently [11].

The absence of global or national agencies that specifically target adolescent health shows the negligence in the policy arena [12]. Investments in adolescent health yield triple dividends, i.e., improved health for the adolescents now, a productive force for tomorrow’s economy, and healthy parents that can reproduce and nurture their children [13]. Undernutrition is the single main contributor to growth retardation worldwide [14]. Therefore, any health care provider that deals with adolescents must take into account their nutrition [15]. A study that compared the nutritional status of adolescents from 200 countries from 1975 to 2016 reported that Ethiopian adolescents had the lowest age-standardized mean BMI for both sexes at 16.8 kg/m^2^ (95% CI, 15.6–17.9 kg/m^2^) for girls and 15.5 kg/m^2^ (95% CI, 14.4–16.6 kg/m^2^) for boys [16]. A study conducted on the impacts of a school feeding program in Ethiopia reported gains in growth indicators such as improved height and body mass index [17]. However, food aid and nutrition-related interventions in Ethiopia, with no exception to the Sidama Zone, often target highly vulnerable segments of the society such as children, nursing mothers, orphans, disabled, elderly, terminally ill patients, HIV victims, and displaced people [18]. Children and elderly are among the primary targets for potential food aid in the Sidama Zone [19]. Research on the subject also appears to focus on one or more of these vulnerable groups, and there are few case studies on the food and nutrition security of adolescents in Ethiopia in general and in the study areas in particular. Therefore, this study targeted adolescents to contribute to the existing knowledge gap concerning the nutritional situations of adolescents in khat- and coffee-growing areas in the Sidama Zone, which is one of the major producers of both crops in Ethiopia [20]. Khat and coffee are prominent cash crops in Ethiopia that support millions of smallholder farmers and earn substantial revenues for the government via taxes and exports.

Ethiopia is the largest coffee producer in Africa and sixth in the world [21]. The Ethiopian coffee sector supports over 15 million people, of which 5 million are smallholder farmers, and coffee is the leading export commodity for the country [22]. Khat is a controversial stimulant plant that is widely grown and consumed in Ethiopia and neighboring countries. It is also a livelihood base for millions of smallholder farmers and many others. Despite its attractive financial returns, khat is often considered an undesirable plant due to its negative health and social impacts [23]. Its buds and leaves contain cathinone and are chewed in fresh condition as a moderate stimulant to alleviate fatigue, increase self-confidence, promote excitation, and suppress sleep and hunger [24]. Khat is often blamed for worsening food insecurity where it grows by displacing conventional staple food crops such as enset (*Ensete ventricosum*) and maize [25]. Enset, or false banana, is a giant herbaceous plant from the Musaceae family with a large underground corm [26]. It is a staple food crop widely grown in Southern Ethiopia, in general, and the study areas, in particular. However, contrary to such claims, our hypothesis is that adolescents from khat-growing areas have improved food security or nutritional status compared to those from coffee-growing areas. This is due to the high economic return of khat compared to coffee and other cash crops.

There are various methods to assess the nutritional status of adolescents, including the use of anthropometry, which is an inexpensive technique to measure the size and composition of a human body [27]. Stunting (height-for-age Z scores < −2 standard deviation (SD) from the median of the reference group) and thinness (body mass index (BMI) for age Z scores < −2 standard deviation (SD) from the median of the reference group) are the commonly used anthropometric indicators to assess the nutritional status of adolescents [5]. Other methods include the use of indirect measurements such as adolescent responses to selected questions adopted from a standardized food insecurity questionnaire, such as the household food insecurity access scale (HFIAS) [28]. Therefore, the main purpose of this study was to assess the food security or nutritional status of adolescents living in selected khat- and coffee-growing areas with three indicators, i.e., stunting, thinness, and a food insecurity experience index (FIE).

## 2. Materials and Methods

### 2.1. Selection of the Study Sites

**Figure 1:**
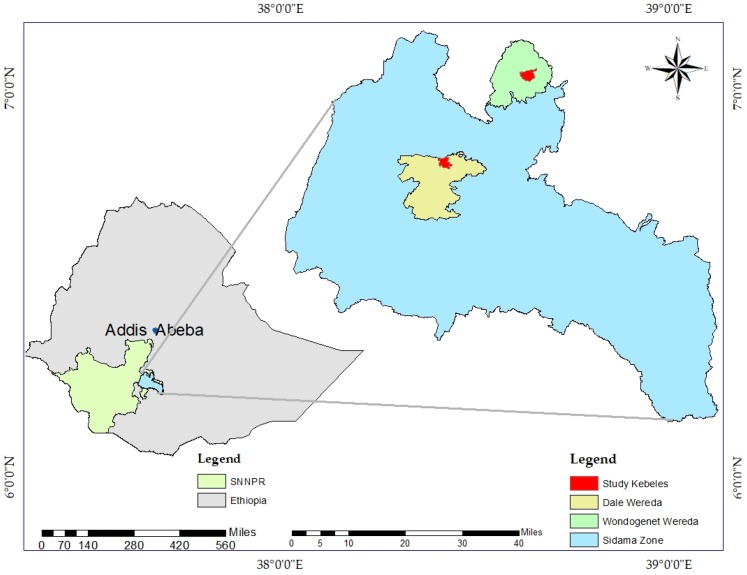
Map of the study areas.

The study sites were selected from the Southern Nations Nationalities and Peoples Regional State (SNNPRS) of Ethiopia by multistage purposive sampling (Figure 1). First, we selected SNNPRS based on the information from the literature and personal experience. Second, we chose the Sidama Zone from 13 administrative zones in the SNNPRS, in consultation with regional experts due to its increasing potential for both khat and coffee. Third, we selected Dale and Wondo Genet woreda (Woreda’ is the local name for district which is the second lowest administrative unit in Ethiopia) from the 19 khat- and coffee- growing woredas in the Sidama zone, in consultation with zonal experts because of their high potential for khat and coffee respectively. Finally, we picked Abaye kebele (Kebele is the local name for peasant association, the lowest administrative unit in Ethiopia) for khat and Chume kebele for coffee from Wondo Genet and Dale, respectively, in consultation with woreda level experts for the same reason. 

#### Description of the Study Sites

The Sidama Zone is the largest producer of both coffee and khat in the SNNPRS and has a total area of 6981.8 km^2^. It lies between the latitudes (5′ 45″ N and 6′ 45″ N) and longitudes (38′ E and 39′ E) with an elevation of 1830–2440 m above sea level [29]. It is one of the densely populated (over 460 persons per km^2^) areas in the region with per household landholding less than two hectares [30]. Wondo Genet is the leading producer of a famous khat known as “beleche”. Similarly, Dale Woreda is a supplier of coffee to regional and national markets. Both of the study areas fall in the subtropical climatic zone with mean annual rainfall of 1123 mm and 1235 mm, mean annual temperature of 17.6 °C and 18.9 °C, and the population of 231,322 and 169,659 for Wondo Genet and Dale, respectively [31]. Chume kebele is located approximately 45 km from Hawassa (Hawassa is the regional capital of the SNNPRS and is located 275 km from the capital Addis Ababa) and has a total area of 800 ha (out of which 426 ha (53.25%) is covered by coffee) and, according to the 2010/11 census, its total population was 5202 [32]. Similarly, Abaye is approximately 27 km from Hawassa and has an area of 1103 ha, out of which 757 ha (68.63%) is covered by perennial crops, primarily khat, and a total population of 11,099 [33].

### 2.2. Methods

We conducted a cross-sectional study in Abaye and Chume from December 2017 to February 2018 to assess the food security of adolescents aged between 12 and 18 years. Though the adolescence age range is between 10–19 years [1], we excluded those below 12 years, in this study, because we assumed that they are too young to adequately comprehend and answer questions about food insecurity experience (FIE). However, the upper age became 18 years by chance, i.e., we used gender-based stratified simple random sampling, and we did not encounter adolescents older than 18 years. The selection was completely random within a stratum (sex category) with no inclusion or exclusion criteria. We selected 234 adolescents (108 from Chume (55 females and 53 males) and 126 from Abaye (62 females and 64 males)) using gender-based stratified random sampling. We collected basic profiles of the adolescents including age in months and recorded their weight and height with standard procedures [34]. Using a digital scale (Seca 881, Germany), we measured the weight to the nearest 0.1 kg. We measured the height to nearest 0.1 cm with a 5-m non-stretchable metallic measuring tape. We locked the tape at 2 m and tied or fixed it to a straight standing object, usually a door frame, and conducted the measurement while the adolescent stood barefoot very close to the tape matching his/her foot to the zero mark and holding their head straight upright. We calculated the body mass index (BMI) of each adolescent by dividing his/her weight in kilogram by the square of his/her height in meters:

(1) $$\mathrm{BMI}[\mathrm{Kg}/m^{2}]=\frac{\mathrm{Weight}\left[\mathrm{Kg}\right]}{{\left(\mathrm{Height}\left[m\right]\right)}^{2}}$$

We checked the distribution of the data and calculated the Z-scores for the height for age (HAZ) and the body mass index for age (BMIZ) using the WHO AnthroPlus software (an open software freely available from the World Health Organization website). We determined the nutritional status of the adolescents using the WHO 2007 growth standards [35]. Accordingly, using the height for age Z scores, we categorized them into three classes of stunting, i.e., non-stunted (HAZ > −2 SD), moderately stunted (HAZ < −2 SD), and severely stunted (HAZ < −3 SD). Similarly, using the BMIZ, we categorized them into thin (BMIZ < −2 SD), severely thin (BMIZ < −3 SD), overweight (BMIZ > 1 SD), and obese (BMIZ > 2 SD). To determine the personal food insecurity experience of the adolescents, not that of their family, we used a four-item index obtained from the sum of the adolescents’ responses to the four questions modified by Hadley and others [28] from the household food insecurity access scale (HFIAS) questionnaire. Accordingly, we instructed each respondent to think about his/her personal experience, not that of his/her household or family, and asked him/her if, in the last three months, he/she had ever:

(i) Worried about having enough food;
(ii) Had to reduce food intake because of shortages of food or money to buy food;
(iii) Had to go without eating because of shortages of food or money to buy food; and
(iv) Had to ask outside the home for food because of shortages of food or money to buy food.

We coded the yes responses 1 and the no responses 0, summed up the scores, and produced a food insecurity experience (FIE) index. Finally, we categorized the respondents as food secure if the sum of the responses for the four questions was 0 and food insecure if the sum was greater than 0. We also asked each adolescent if he/she had been sick (unable to go to school, work, or visited a doctor) from any illness in the last 30 days and coded yes responses 1 and the no responses 0.

To ensure ethical standards, we obtained informed consent prior to any measurement or interview from the participating adolescents and their parents. We obtained ethical clearance (ID: 15–186) from the University of Tokyo under the food insecurity impacts of the industrial crops expansion in the sub-Saharan Africa (FICESSA) project. Therefore, only willing adolescents participated in the study and their anthropometric data and responses to the interview questions were treated with confidentiality.

### 2.3. Statistical Analyses

We used Microsoft Excel to compute descriptive statistics, such as the percent, mean, and median, and SPSS (Version 23.0; IBM Corp., Armonk, NY, USA) to conduct statistical tests (e.g., the chi-squared test). We conducted binary and multivariate logistic regression analyses using SPSS (version 23) to test potential associations between each of the three dependent variables (stunting, thinness, and food insecurity experience) and the 11 explanatory variables. First, we conducted bivariate analyses with each of the independent variables, by entering a single independent variable and a dependent variable at a time. Then, we ran multivariate logistic regression analyses to select covariates for each of the dependent variables with the stepwise forward selection method [36]. The variables we included in the multivariate logistic regression analyses are those that have shown significant association in the bivariate analyses and others reported in the literature [37].

## 3. Results

### 3.1. Descriptive Results

Table 1 presents the socio-demographic characteristics of the sample adolescents. The total number of sample adolescents was 234 (117 girls and 117 boys), of which 126 (62 girls and 64 boys) were from Abaye (a khat-growing area) and 108 (55 girls and 53 boys) are from Chume (a coffee-growing area). In terms of livelihood, 73% of the adolescents from Abaye were from families that depend on khat, 5.6% of them were from families that rely on coffee, and 21.4% of them were from families that earned their living from other means of livelihood, such as farming mixed crops, employment, and small businesses. Similarly, 60.2% and 39.8% of the adolescents from Chume were from families that primarily rely on coffee and other means of livelihood, respectively. The girls from Chume are younger and have shorter median height and lower HAZ-score than girls from Abaye; however, boys have comparable age, height, and HAZ-score regardless of the area (Table 1). Overall, the mean Z-scores of height for age of the girls and body mass index for age for both sexes from Chume were significantly lower than the corresponding values from Abaye (Table 1).

### 3.2. The Nutritional Status and Food Security of the Adolescents

#### 3.2.1. Nutritional Status: Stunting and Thinness

**Figure 2:**
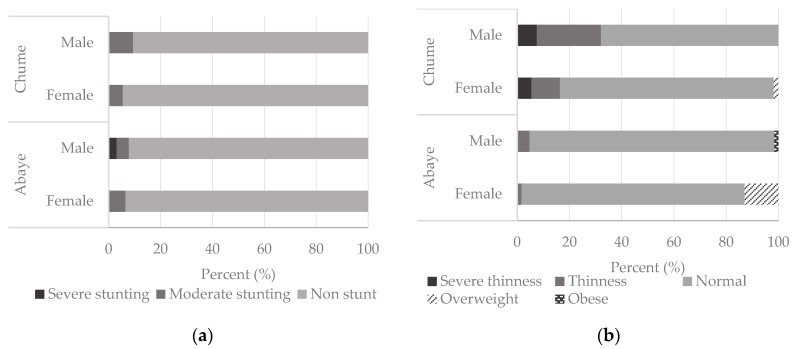
Adolescent (**a**) stunting and (**b**) thinness.

**Figure 3:**
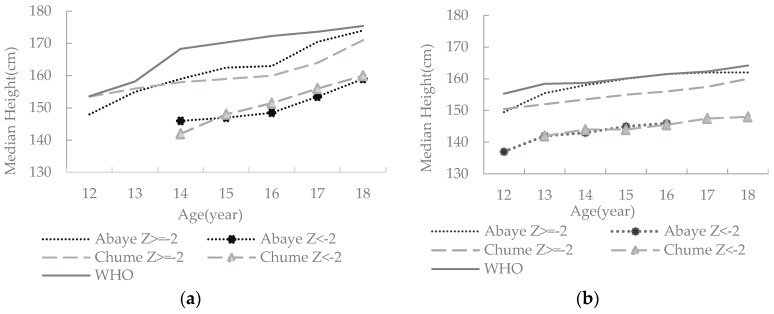
Height of stunted and non-stunted adolescent (**a**) boys and (**b**) girls by age.

The prevalence of stunting appears low, i.e., only 17 (7.3%) of all adolescents were stunted. We observed severe stunting for 3.1% of the boys from Abaye. Area-wise, 6.5% and 5.5% of the girls and 7.8% and 9.4% of the boys were stunted in Abaye and Chume, respectively (Figure 2a). However, there was no significant difference in the stunting of adolescents between study sites (χ^2^ = 1.530, *p* = 0.216) and genders (χ^2^ = 0.153, *p* = 0.696). The median height for age of all the stunted adolescents was below the corresponding median height for age of the non-stunted adolescents and that of the WHO 2007 growth reference data, regardless of the area or gender (Figure 3). The mean height of the stunted girls was 144.1 cm, which was 11.8 cm shorter than the mean height of the non-stunted girls. Similarly, the mean height of the stunted boys was 151.1 cm, which was 10.6 cm shorter than the mean height of the non-stunted boys.

The overall prevalence of thinness 30 (12.8%) in the study areas was high. Nevertheless, it was significantly higher in Chume than in Abaye (χ^2^ = 22.726, *p* = 0.000) and for boys compared to girls (χ^2^ = 5.506, *p* = 0.019). We observed severe thinness for 4 (7.5%) boys and 3 (5.5%) girls from Chume, while none of the adolescents from Abaye were severely thin (Figure 2b). The prevalence of thinness was 1 (1.6%) and 9 (16.4%) for the girls and 3 (4.7%) and 17 (32.1%) for the boys in Abaye and Chume, respectively. Moreover, 8 (12.9%) and 1 (1.8%) of the girls were overweight in Abaye and Chume, respectively, while only 1 (1.6%) boy from Abaye was obese.

#### 3.2.2. Food Insecurity Experience (FIE)

Of the adolescents, 89 (38.0%) reported food insecurity experiences. Accordingly, 40.3% and 30.9% of the girls and 45.3% and 34.0% of the boys were food insecure in Abaye and Chume, respectively. The proportion of adolescents that experienced food insecurity did not appear to differ across sites (χ^2^ = 2.694, *p* = 0.101) or gender (χ^2^ = 0.453, *p* = 0.501).

### 3.3. Factors Associated with the Stunting, Thinness, and FIE of Adolescents

Multivariate logistic regression analyses revealed that adolescent age and maternal education are significantly associated with stunting (Table 2). The odds of stunting for adolescents aged 12–14 years is 3.569 times (adjusted OR = 3.569; 95% CI, 1.109, 11.485) greater than that of those aged 15–18 years, all things being equal. Similarly, the odds of adolescents who have illiterate mothers (who cannot read and write properly) of being stunted is 5.641 times (adjusted OR = 5.641; 95% CI, 1.560, 20.392) greater than that of those who have literate mothers.

As shown in Table 3, gender, area or place of residence, the number of meals a day, and livelihood show significant associations with the adolescents’ thinness. Controlling for other variables, the odds of thinness for girls is 0.386 times (adjusted OR = 0.386; 95% CI, 0.157, 0.944) smaller than that of boys, and the odds of thinness for adolescents from Abaye is 0.123 times (Adjusted OR = 0.123, 95% CI, (0.040, 0.375) smaller than that of adolescents from Chume. Likewise, adolescents from families whose livelihoods depend on khat are 0.119 times (adjusted OR = 0.119; 95% CI, 0.035, 0.401) less likely to be thin than those who are from families that earn their living from coffee. Finally, the odds of thinness for adolescents who said they eat less than three meals a day is 4.164 times (adjusted OR = 4.164; 95% CI, 1.629, 10.647) greater than those who claimed to eat three or more meals a day.

The food insecurity experiences (FIEs) of adolescents were associated with health problems in the past 30 days (Table 4). The odds of experiencing at least one of the four food insecurity indicators was 0.235 times (adjusted OR = 0.235; 95% CI, 0.115, 0.483) smaller for those adolescents who had a health problem in the past 30 days compared to those who did not have such a problem in the stated period.

## 4. Discussion

The overall rate of stunting (7.3%) and thinness (12.8%) reported in this study are within the WHO’s prevalence range of low and high, respectively [38]. Previous studies have associated adolescents’ nutritional status with a myriad of personal characteristics (e.g., genetic, age, etc.), household variables (e.g., income, family size, etc.), and environmental factors, like hygiene [39]. Accordingly, we associate the observed differences in adolescents’ nutritional status in this study with two groups of factors: (1) personal characteristics and (2) socioeconomic (e.g., parental education) and environmental factors.

### 4.1. Personal Characteristics

Many genetic and environmental factors influence human growth characteristics, such as height, in addition to nutrition [40]. For example, genetic factors, such as race, account for 60–80% of the height differences between individuals [41]. Hence, we argue that at least part of the observed deviations from the WHO 2007 standard reference is likely due to genetic differences (e.g., race) between our sample groups and that of the WHO reference samples. In this study, younger adolescents (12–14 years) have a higher risk of stunting than older ones (15–18 years), and this is in agreement with previous studies, such as Mulugeta and others [42], from the Tigray region in Northern Ethiopia, and Barman [43] from India. Thinness was significantly higher for the boys than the girls, especially for those from Chume. This gendered difference could be due to the boys’ tendencies to engage in energy-intensive outdoor activities, like working on agricultural farms and playing soccer [44], whereas girls, who are customarily close to home and assist their mothers with household works, including food preparation, are likely to have better access to food than boys. The higher prevalence of thinness among boys is in line with the national trend, i.e., the national average rate of thinness for boys aged 10–19 years is nearly double of that of girls of the same age group [3]. However, this result contradicts some of the previous studies in Ethiopia, such as Tessema [45], and Hadley and others [28], have reported a gender bias that favors boys in intra-household food allocations during scarcities. The observed contradiction would be due to differences in socioeconomic factors (e.g., ethnicity, culture, etc.) between the study sites [46].

### 4.2. Socioeconomic and Environmental Factors

The prevalence of stunting in the study areas appears low (less than 10%) and does not vary significantly across the study sites. However, non-stunted adolescents from Abaye appear to have relatively better height than those from Chume (Figure 3). The overall prevalence of thinness (12.8%) is high by the WHO standard [38] and is disproportionately very high in Chume for both sexes. Nonetheless, they are less than the national prevalence rates (29% for girls and 59% for boys) for adolescents in Ethiopia [3]. We contend that the relatively smaller prevalence of thinness and better linear growth (see Figure 3, and Figures S1–S4 in the Supplementary Materials) of adolescents from Abaye are in accordance with our hypothesis. This is likely due to (1) better household income from khat (the livelihood base for the majority of the adolescents’ families in Abaye); (2) the presence of rich agroforestry systems; and (3) access to better infrastructure.

Khat, as a cash crop, outcompetes other crops, including coffee, primarily due to its fast growth (2 to 3 times harvests per year), stable and lucrative income, and better resistance to shocks (e.g., drought, price fluctuation) [47]. For example, a khat farm on a 0.1 ha plot in Wondo Genet can generate 25 times and six times more income than maize and sugarcane, respectively [25]. However, despite its attractive income, some blame khat for exacerbating food insecurity by displacing staple food crops and disempowering women [25,48]. Notwithstanding the dominance of men over large income streams (especially when the khat is sold at once on a farm), there are also cases where women harvest small quantities of khat and sell it on the retail market to buy food and other utilities. In addition, khat has no defined growing and harvesting seasons, especially where there is adequate moisture (e.g., irrigation) and, therefore, can generate income every three to four months. There are pieces of evidence that khat improves the overall livelihood, including food security, of its growers by generating fast and substantial income [20]. Another reason for the better nutritional status in Abaye would be the presence of rich agroforestry systems in the area [49]. Well managed agroforestry systems are sources of quality fruits, vegetables, and fiber and, hence, improve food security [50]. Proximity to the large towns, like Hawassa and Shashemene, and better infrastructure (e.g., roads, irrigation) would also give better opportunities (e.g., access to off-farm jobs, buy foods at reasonable prices, etc.) for the Abaye residents to improve their food security [51].

Coffee, the main livelihood for many families in Chume, is highly susceptible to various shocks (e.g., frost, pests and diseases, and price drops), and its market is heavily regulated by the government [52]. Farmers in the study areas often complain about the disappointingly low coffee prices and some of them are even switching to other crops such as khat and eucalyptus [23]. Coffee harvests frequently fail in the study areas, and expose its growers to a higher risk of food insecurity [53]. In the event of such shocks, farmers try to cope by selling their assets (e.g., cattle to buy food) or use other means, like reducing the amount of food intake, which could eventually deplete their resilience capacity [52]. Moreover, unlike Abaye, Chume is relatively far from infrastructure (e.g., there is no irrigation water) and it is one of the beneficiaries of the government’s productive safety net program (PSNP), a program that provides food in exchange of public work for extremely poor citizens. Therefore, the observed high prevalence of thinness in Chume could be due to low returns from coffee and fewer opportunities for residents.

The staple foods in both of the study areas are from enset and maize. Although they are good sources of carbohydrate and quality fiber, popular enset-based foods like “kocho” and “bulla” are poor in protein and vitamins and lack essential nutrients [54]. As a result, too much dependence on such foods would compromise one’s nutritional status. Insufficient carbohydrate and nutrient intake, low diet diversity, and frequent meal skipping practices increase the risk of stunting and thinness [55,56].

Several other studies have reported associations between adolescents’ nutritional status and socioeconomic and environmental factors [37,57]. A comparative study conducted on Indian adolescents and adolescents of Indian origin from the United Arab Emirates (UAE) has reported a higher prevalence of stunting and thinness in the Indian group living in India and attributed it to the poor living conditions in India [58]. Another study from Canada reported that youths from low-income households had lower height percentiles, consumed less milk, and had higher levels of nutritional deprivation [59]. Similarly, many studies from Ethiopia have reported positive associations between adolescents’ poor nutritional status (e.g., stunting, thinness) and various socioeconomic factors, such as low household income, large family size, poor sanitation, and low maternal education [37,42,60]. According to Demissie [61] and Sandifordt [62], literate mothers (who can properly read and write) can easily adopt technical supports and health packages provided by the extension workers and would improve the nutritional status of their children and families.

Our study has revealed important findings which would inform pertinent policies not only in Ethiopia, but also in other khat- and coffee-growing countries, like Kenya, Uganda, and Madagascar [20,63]. Its strengths lie in its focus both in choosing the target group (adolescents) and the study sites (areas with the two competing cash crops), and the use of three different food security indicators. However, because it is a cross-sectional study from only two sites, its results should not be generalizable beyond the stated areas, times, and ages of the adolescents.

## 5. Conclusions

The study has revealed that food insecurity exists among adolescents in both sites but is less severe in the khat-growing region of Abaye than in the coffee-growing region of Chume, and less severe in girls than boys. Additional in-depth studies are needed to better understand the adolescents’ food security in these and other khat- and coffee-growing areas in Ethiopia. Possible interventions for adolescents in coffee-growing areas should include increasing the low coffee prices, introducing targeted school feeding programs for adolescents, and educating mothers on the importance of proper nutrition.

## Figures and Tables

**Table 1:** Socio-demographic characteristics of the sample adolescents from the Sidama Zone, Southern Ethiopia.

Variable	Area	Sex	N	Min	Max	Median	Mean (SD)	S. Error	d.f	Test Statistics ^1^	*p*-Value
Age (year)	Abaye	F	62	12.0	18.0	14.4	14.72 (2.05)	0.26			
	Chume		55	12.0	17.8	13.9	13.90 (1.82)	0.25	1	1470	0.191
	Abaye	M	64	12.0	18.0	14.7	14.95 (2.03)	0.25			
	Chume		53	12.1	18.0	14.9	15.00 (1.61)	0.28	1	1113.5	0.001
Weight (kg)	Abaye	F	62	30	71	50.0	48.72 (9.41)	1.21			
	Chume		55	28	69	43.0	42.80 (7.18)	0.97	1	981	0.000
	Abaye	M	64	29	67	48.0	46.31 (9.81)	1.22			
	Chume		53	31	68	43.0	45.76 (9.16)	1.26	1	1644	0.776
Height (cm)	Abaye	F	62	137	173	157.5	155.95 (7.92)	1.00			
	Chume		55	143	173	154.0	154.36 (6.59)	0.89	1	1432	0.136
	Abaye	M	64	137	180	158.5	157.81 (10.27)	1.28			
	Chume		53	144	188	162.0	163.68 (9.41)	1.29	1	1165.5	0.004
HAZ	Abaye	F	62	−2.73	1.71	−0.25	−0.33 (0.91)	0.12			
	Chume		55	−2.73	1.52	−0.91	−0.82 (0.94)	0.13	1	1217.5	0.008 **
	Abaye	M	64	−3.46	1.64	−0.37	−0.55 (1.05)	0.13			
	Chume		53	−2.63	2.24	−0.53	−0.50 (1.15)	0.16	1	1505.5	0.297
BMIZ	Abaye	F	62	−2.09	1.82	0.09	−0.01 (0.85)	0.11			
	Chume		55	−4.07	1.01	−0.77	−0.96 (1.04)	0.14	1	831	0.000 **
	Abaye	M	64	−2.29	2.67	−0.41	−0.43 (0.90)	0.11			
	Chume		53	−3.32	0.04	−1.43	−1.60 (0.86)	0.12	1	866.5	0.000 **
Education (school year)	Abaye	F	62	2	12	5	5.94 (2.78)	0.35			
	Chume		55	2	10	7	6.42 (2.20)	0.29	1	1410	0.102
	Abaye	M	64	0	10	6	5.64 (2.22)	0.28			
	Chume		53	4	10	6	6.02 (1.69)	0.23	1	1577.5	0.511
Family size (number)	Abaye	F	62	2	14	7.5	7.29 (2.51)	0.319			
	Chume		55	3	9	6	6.24 (1.41)	0.191	1	1159.5	0.003 **
	Abaye	M	64	3	15	7.5	7.33 (2.25)	0.281			
	Chume		53	3	8	6	5.94 (1.34)	0.184	1	971.5	0.000 **

^1^ Mann–Whitney U, ** the difference is significant (*p* < 0.01).

**Table 2:** Multivariate analyses of risk factors for stunting of adolescents in the Sidama Zone, Southern Ethiopia.

	*N* (%)		Crude OR	(Sig.)	Adjusted OR	(Sig.)
Variable	Not Stunted	Stunted	(95% CI)	*p*-Value	(95% CI)	*p*-Value
**Sex**						
Female	110 (94.0)	7 (6.0)	0.681 (0.250, 1.854)	0.452	0.616 (0.192, 1.971)	0.414
Male	107 (91.5)	10 (8.5)	1.00		1.00	
**Age**						
12–14 years	107 (89.2)	13 (10.8)	3.341 (1.056, 10.571)	0.04*	3.569 (1.109, 11.485)	0.033 *
15–18 years	110 (96.5)	4 (3.5)	1.00		1.00	
**Education**						
Up to grade 6	89 (93.7)	6 (6.3)	0.784 (0.280, 2.199)	0.644	0.364 (0.100, 1.318)	0.124
Above grade 6	128 (92.1)	11 (7.9)	1.00		1.00	
**Maternal Education**						
Illiterate ^**@**^	101 (87.8)	14 (12.2)	5.360 (1.497, 19.184)	0.01*	5.641 (1.560, 20.392)	0.008 **
Literate	116 (97.5)	3 (2.5)	1.00		1.00	
**Area**						
Abaye	117 (92.9)	9 (7.1)	0.962 (0.358, 2.585)	0.938	0.993 (0.256, 3.855)	0.992
Chume	100 (92.6)	8 (7.8)	1.00		1.00	
**Family size**						
Over 6 members	117 (90.7)	12 (9.3)	2.051 (0.699, 6.022)	0.191	3.196 (0.954, 10.704)	0.060
Up to 6 members	100 (95.2)	5 (4.8)	1.00		1.00	
**Off farm income**						
Yes	67 (89.3)	8 (10.7)	1.99 (0.736, 5.382)	0.175	2.337 (0.766, 7.133)	0.136
No	150 (94.3)	9 (5.7)	1.00		1.00	
**Health problem ^Ф^**						
Yes (in 30 days)	67 (90.5)	7 (9.5)	1.567 (0.572, 4.294)	0.382	2.142 (0.653, 7.022)	0.209
No (in 30 days)	150 (93.8)	10 (6.2)	1.00		1.00	
**Number of meals**						
<3 meals a day	87 (89.7)	10 (10.3)	2.135 (0.783, 5.822)	0.139	1.664 (1.090, 2.290)	0.457
≥3 meals a day	130 (94.9)	7 (5.1)	1.00		1.00	
**Animal protein**						
<3 times a week	46 (90.2)	5 (9.8)	1.549 (0.519, 4.620)	0.433	1.484 (0.420, 5.245)	0.540
≥3 times a week	171 (93.4)	12 (6.6)	1.00		1.00	
**Family livelihood**						
Khat	85 (92.4)	7 (7.6)	1.894 (0.472, 7.599)	0.368	1.924 (0.213, 17.353)	0.852
Other ^**Θ**^	63 (90.0)	7 (10.0)	2.556 (0.633, 10.311)	0.187	2.266 (0.153, 4.701)	0.560
Coffee	69 (95.8)	3 (4.2)	1.00		1.00	

^**@**^ Cannot read or write; * the difference is significant (*p* < 0.05); ** the difference is significant (*p* < 0.01). ^**Ф**^ Refers to any illness that had forced an adolescent to miss classes or absent from work or visit a doctor in the last 30 days. ^**Θ**^ Refers to means of livelihoods other than khat and coffee like mixed-crop farming, employment, business, social support, etc.

**Table 3:** Multivariate analyses of risk factors for the thinness of adolescents in the Sidama Zone, Southern Ethiopia.

	*N* (%)		Crude OR	(Sig.)	Adjusted OR	(Sig.)
Variable	Not Thin	Thin	(95% CI)	*p*-Value	(95% CI)	*p*-Value
**Sex**						
Female	108 (92.3)	9 (7.7)	0.381 (0.166, 0.872)	0.022 *	0.386 (0.157, 0.944)	0.037 *
Male	96 (82.1)	21 (17.9)	1.00		1.00	
**Age**						
12–14 years	99 (86.8)	15 (13.2)	1.061 (0.493, 2.283)	0.880	1.173 (0.439, 3.133)	0.751
15–18 years	105 (87.5)	15 (12.5)	1.00		1.00	
**Education**						
Up to grade 6	119 (85.6)	20 (14.4)	1.429 (0.636, 3.207)	0.387	1.657 (0.607, 4.523)	0.325
Above grade 6	85 (89.5)	10 (10.5)	1.00		1.00	
**Maternal Education**						
Illiterate ^**@**^	91 (79.1)	24 (20.9)	4.967 (1.948, 12.668)	0.001 **	1.373 (0.401, 4.700)	0.613
Literate	113 (95.0)	6 (5.0)	1.00		1.00	
**Area**						
Abaye	122 (96.8)	4 (3.2)	0.103 (0.035, 0.307)	0.000 **	0.123 (0.040, 0.375)	0.000 **
Chume	82 (75.9)	26 (24.1)	1.00		1.00	
**Family size**						
Up to 6 members	89 (84.8)	16 (15.2)	1.477 (0.685,3.186)	0.320	1.394 (0.263, 1.588)	0.341
Over 6 members	115 (89.1)	14 (10.9)	1.00		1.00	
**Off farm income**						
Yes	67 (89.3)	8 (10.7)	0.744 (0.315, 1.758)	0.500	0.889 (0.264, 2.989)	0.849
No	137 (86.2)	22 (13.8)	1.00		1.00	
**Health problem ^Ф^**						
Yes (in 30 days)	64 (86.5)	10 (13.5)	1.094 (0.484, 2.470)	0.829	0.883 (0.350, 2.226)	0.792
No (in 30 days)	140 (87.5)	20 (12.5)	1.00		1.00	
**Number of meals**						
<3 meals a day	74 (76.3)	23 (23.7)	5.772 (2.364, 14.097)	0.000 **	4.164 (1.629, 10.647)	0.003 **
≥3 meals a day	130 (94.9)	7 (5.1)	1.00		1.00	
**Animal protein**						
<3 times a week	43 (84.3)	8 (15.7)	1.362 (0. 567, 3.271)	0.490	1.233 (0.405, 3.751)	0.712
≥3 times a week	161 (88.0)	22 (12.0)	1.00		1.00	
**Family livelihood**						
Khat	88 (95.7)	4 (4.3)	0.127 (0.041, 0.393)	0.000 **	0.119 (0.035, 0.401)	0.001 **
Other ^**Θ**^	63 (90.0)	7 (10.0)	0.310 (0.121, 0.794)	0.015 *	0.314 (0.096, 1.029)	0.056
Coffee	53 (73.4)	19 (26.4)	1.00		1.00	

^**@**^ Cannot read or write; * the difference is statistically significant (*p* < 0.05); ** the difference is statistically significant (*p* < 0.01). ^**Ф**^ Refers to any illness that had forced an adolescent to miss classes or absent from work or visit a doctor in the last 30 days. ^**Θ**^ Refers to means of livelihoods other than khat and coffee like mixed-crop farming, employment, business, social support, etc.

**Table 4:** Multivariate analyses of risk factors for adolescent food insecurity experiences (FIE) in the Sidama Zone, Southern Ethiopia.

	*N* (%)		Crude OR	(Sig.)	Adjusted OR	(Sig.)
Variable	Food Secure	Food Insecure	(95% CI)	*p*-Value	(95% CI)	*p*-Value
**Sex**						
Female	75 (64.1)	42 (35.9)	0.834 (0.492, 1.415)	0.501	0.787 (0.445, 1.393)	0.412
Male	70 (59.8)	47 (40.2)	1.00		1.00	
**Age**						
12–14 years	76 (63.3)	44 (36.7)	0.888 (0.524, 1.505)	0.658	0.707 (0.368, 1.357)	0.298
15–18 years	69 (60.5)	45 (39.5)	1.00		1.00	
**Education**						
Up to grade 6	55 (57.9)	40 (42.1)	1.336 (0.782, 2.282)	0.289	1.480 (0.766, 2.858)	0.244
Above grade 6	90 (64.7)	49 (35.3)	1.00		1.00	
**Maternal Education**						
Illiterate ^**@**^	76 (66.1)	39 (33.9)	0.708 (0.417, 1.204)	0.202	0.625 (0.286, 1.365)	0.239
Literate	69 (68.0)	50 (42.0)	1.00		1.00	
**Area**						
Abaye	72 (57.1)	54 (42.9)	1.564 (0.916, 2.673)	0.102	1.244 (0.466, 3.320)	0.663
Chume	73 (67.6)	35 (32.4)	1.00		1.00	
**Family size**						
Up to 6 members	78 (61.5)	51 (39.5)	1.153 (0.677, 1.962)	0.600	0.903 (0.498, 1.639)	0.738
Over 6 members	67 (63.8)	38 (36.2)	1.00		1.00	
**Off farm income**						
Yes	41 (54.7)	34 (45.3)	1.568 (0.896, 2.745)	0.115	1.312 (0.625, 2.756)	0.473
No	104 (65.4)	55 (34.6)	1.00		1.00	
**Health problem ^Ф^**						
Yes (in 30 days)	58 (78.4)	16 (21.6)	0.329 (0.174, 0.620)	0.001**	0.235 (0.115, 0.483)	0.000 **
No (in 30 days)	87 (54.4)	73 (45.6)	1.00		1.00	
**Number of meals**						
<3 meals a day	64 (63.9)	35 (36.1)	0.868 (0.507, 1.486)	0.605	1.201 (0.564, 2.557)	0.634
≥3 meals a day	83 (60.6)	54 (39.4)	1.00		1.00	
**Animal protein**						
<3 times a week	38 (74.5)	13 (25.5)	0.482 (0.240, 0.965)	0.039 *	0.651 (0.305, 1.392)	0.269
≥3 times a week	107 (58.5)	76 (41.5)	1.00		1.00	
**Family livelihood**						
Khat	55 (59.8)	37 (40.2)	1.634 (0.847, 3.152)	0.143	1.302 (0.439, 3.859)	0.634
Other ^**Θ**^	39 (55.7)	31 (44.3)	1.930 (0.965, 3.861)	0.063	1.608 (0.686, 3.768)	0.275
Coffee	51 (70.8)	21 (29.2)	1.00		1.00	

^**@**^ Cannot read and write properly; * the difference is statistically significant (*p* < 0.05); ** the difference is statistically significant (*p* < 0.01). ^**Ф**^ Refers to any illness that had forced an adolescent to miss classes or absent from work or visit a doctor in the last 30 days. ^**Θ**^ Refers to means of livelihoods other than khat and coffee like mixed-crop farming, employment, business, social support, etc.

## Supplementary Materials

The following are available online at http://www.mdpi.com/2072-6643/10/8/980/s1, Figure S1: Height for age (HAZ) distribution of 126 (male = 64, female = 62) adolescents aged 12–18 years from Abaye, southern Ethiopia, along with the WHO 2007 growth reference., Figure S2: Height for age (HAZ) distribution of 108 (male = 53, female = 55) adolescents aged 12–18 years from Chume, southern Ethiopia, along with the WHO 2007 growth reference., Figure S3: Body mass index for age (BMIZ) distribution of 126 (male = 64, female = 62) adolescents aged 12–18 years, from Abaye, southern Ethiopia, along with the WHO 2007 growth reference., Figure S4: Body mass index for age (BMIZ) distribution of 108 (male = 53, female = 55) adolescents aged 12–18 years, from Chume, southern Ethiopia, along with the WHO 2007 growth reference.
